# Medical Literature and Medical Journals

**Published:** 1883-02

**Authors:** 


Medical Literature and Medical Journals.—The results of thought and experiment in the domain of medicine, obtained for it by the work of any one man or of any set of men, are looked upon as common property by the rest of the profession. That is, a book that represents the work of a life time—Sir Thomas Watson's or Frerichs', for instance—is regarded as a fountain to which all may go and draw thence what facts or ideas they deem of highest value.

Thus the offspring of one man's thought creeps into general literature, sooner or later to lose its individuality, or, rather, to become axiomatic. Anything good belongs to all alike. Any progress in medicine in one part of the world is heralded on all sides, repeated, modified, used, and then merged into the great mass of facts. This is as it should be: it shows, we think, that there is a broad, cosmopolitan and generous spirit—generosity in the donor, generosity in the science that so unhesitatingly receives the good. Still, some one must be *first* to announce that work or thought of merit, for it cannot of itself make way into the mind of man. And here is where the rivalry of nation, man and journal strives to keep in the front rank. It is, or should be, always a matter of pride with journals—with medical journals especially—to be *first* in propagating welcome, helpful and meritorious news to their readers. Now, when the same article is sent to several journals *simultaneously*, when it is published by each—and each ignorant of its publication elsewhere—who shall claim priority? None can of a certainty. There is no honor for the publisher in such a case; and it does not seem in good taste, indeed scarcely in good faith, for a contributor to anticipate his manuscript in this way. Once an article is published in one paper it is carried everywhere, if deserving of such honor, and recognition is always made of the original source. If a man has anything good to state, it will make its way uncared for after the first publication; he will find it in all out-of-the-way corners and in publications that he never wot of. But when several copies of the same thing are despatched to as many journals, does it not seem that there is a lurking fear that as this is an only chance the most must be made of it? The sender feels sure that three or four will publish it; and should it go no further, but merely "stick there," he is satisfied.

This may be a pessimistic view; but it certainly is a milder one than would be taken of it by magazines devoted to general literature. And we are led in this connection to remark that contributions to medical literature are not made often enough by those who have large experience or who read extensively. Their duty is to build up a medical literature that shall be on a par with that of our trans-Atlantic brethren. On looking over a foreign medical book one is struck with the great frequency with which reference is made to articles in journals. Virchow, Jenner, Quincke, Charcot, Frerichs—these men and a host of others are constantly found in the literature of their respective countries, as represented by the journals, and yet we must believe that they are as busy as the majority of American practitioners. The fact is, they have a habit of writing, and the habit grows, while the tendency among us, if it exists, is very slow in its development, and many instructive and interesting cases and views are thus lost that would otherwise become part and parcel of our medical knowledge.

